# Identifying and Addressing Barriers to Live Primary Prostate Cancer Cell Research in Veterans

**DOI:** 10.1158/2767-9764.CRC-25-0776

**Published:** 2026-06-29

**Authors:** Alex Carlson, Erika Heninger, Emma E. Recchia, Joel Pollen, Jennifer Piccolo, S. Carson Callahan, Shuang G. Zhao, David Kosoff

**Affiliations:** 1 Department of Medicine, University of Wisconsin-Madison, Madison, Wisconsin.; 2 Carbone Cancer Center, University of Wisconsin-Madison, Madison, Wisconsin.; 3 William S. Middleton Memorial Veterans Hospital, Madison, Wisconsin.; 4 Department of Human Oncology, University of Wisconsin-Madison, Madison, Wisconsin.

## Abstract

Increased comorbidities and unique service-related exposures among veterans may contribute to unique tumor biology that can influence tumor behavior and response to treatment. Research that incorporates live cells from veteran donors can support investigation of the genomic and transcriptomic biology of veteran tumors as well as how those molecular alterations affect tumor function, such as growth, metastasis, and treatment sensitivity. We conducted a systematic literature review to assess veteran-focused prostate cancer studies published in the last decade that incorporate live primary cells. We found that in the past 10 years, 105 prostate cancer studies were published that utilized live primary cells from the general population compared with zero studies utilizing live primary cells from veterans. Analysis of veterans enrolled in a study utilizing live primary cells at the William S Middleton Veterans Affairs demonstrated that 94% of veterans who were presented with a live cell biospecimen donation study ultimately enrolled. Furthermore, survey responses from veterans enrolled in this study showed that 83% of veterans found their research engagement to be a meaningful experience and 70% reported increased healthcare satisfaction as a result of their participation. These findings suggest that the lack of live cell prostate cancer research in veterans seems to be due to a lack of research opportunities for veterans and not a lack of veteran interest in participation Finally, we demonstrate that implementation of an informatics-based patient screening strategy can potentially support more veteran-focused studies by reducing participant screening time by 89%.

**Significance:**

Live primary cells are a unique model for prostate cancer research that provides both molecular and functional readouts to support advances in veteran prostate cancer care. We detail the shortage of live primary research in veterans and address potential barriers to future studies incorporating this model.

## Introduction

Although prostate cancer is a disease that can affect all men, military veterans (veterans) are diagnosed with prostate cancer at nearly double the rate of the general population, with more of their cancers discovered at advanced or incurable stages (1, 2). Veterans also have a distinct array of cancer risk factors, including larger numbers of health comorbidities, higher rates of cancer-associated behaviors, and a variety of unique carcinogenic exposures (e.g., Agent Orange), which have been associated with prostate cancer incidence, mortality, and adverse outcomes (3–5). These differences in both prostate cancer epidemiology and cancer-associated risk factors suggest that more research is needed to understand the biology of veteran prostate cancer and determine whether veteran prostate cancers should be screened, diagnosed, or treated differently than prostate cancers in the general population.

Patient-derived biospecimens, such as blood and tissue, provide the foundation for translationally relevant model systems and are essential tools for prostate cancer discovery. Primary cells from both blood and tissue directly reflect the molecular and functional biology of a patient and can be used to characterize the molecular landscape of a patient’s cancer, estimate prognosis, and predict treatment response (6, 7). Inclusion of veteran-derived biospecimens into prostate cancer studies can therefore support the investigation of the biologic pathways that drive cancer in veterans as well as guide research advances with direct applicability to the care of veterans with prostate cancer. Fortunately, recent breakthroughs in technology have enabled high-throughput, cost-effective, and comprehensive analysis of the molecular biology of primary tumors (8). These advances have supported significant advancement in biospecimen-based cancer research, including within the Veterans Affairs (VA) system, which has pioneered numerous initiatives, such as the Million Veterans Project, National Precision Oncology Program, Lung Precision Oncology Program, and Precision Oncology Program for Cancer of the Prostate (POPCaP), which leverage veteran donor biospecimens to advance veteran-focused biomedical research (9–13).

The increase in biospecimen-focused research within the VA has thus far relied heavily on the utilization of archived, formalin-fixed paraffin-embedded (FFPE) biospecimens (14–17). The advantage of fixed biospecimens is that they provide a readily accessible source of large quantities of patient tissue samples that can be used to investigate genomic and transcriptomic alterations as well as protein expression and morphologic features of large tumor cohorts (18). The information gained from the analysis of FFPE tissue archives can be correlated with clinical data to identify epidemiologic associations and to develop novel biomarkers (15, 19, 20). However, a key limitation of fixed biospecimens is that they cannot be utilized for investigation of functional endpoints in live assays. Specifically, these specimens cannot be used to study prostate tumor cell growth, invasion, cell–cell communication, or treatment sensitivity. Furthermore, FFPE models are not suitable to determine whether and how molecular alterations contribute to early prostate tumor initiation, progression, and therapeutic resistance. Fixed biomaterial also has limited utility for the investigation of new treatment strategies to improve veteran outcomes. To answer these questions, researchers need live primary cells that are derived from veteran biospecimens and can be utilized to investigate both molecular and biologic endpoints (18).

Given the potential for live primary cell assays to advance veteran prostate cancer research and care, we performed a thorough literature review to investigate the number of basic and translational prostate cancer research studies that utilized live cells derived from veteran biospecimens. This analysis found no studies published in the last 10 years that met these criteria compared with 105 published studies that utilized live primary cells in the general population. Through a survey-based analysis of veterans enrolled in a translational prostate cancer research study conducted at the William S Middleton Veterans’ Hospital in Madison, Wisconsin (Madison VA), we found that veterans at our site were highly interested in contributing biospecimens for live cell assays. We therefore developed and assessed a pilot informatics-based strategy specifically designed to limit key barriers for investigators seeking to enroll veterans in research studies, such as patient screening and review of donor eligibility criteria.

## Materials and Methods

### Database search strategy

Literature review of published data on “veteran prostate cancer research” involving “primary cell culture” was performed independently by two researchers in a stepwise approach. The analysis was performed using the PubMed (RRID: SCR_004846) and Embase (RRID: SCR_001650) databases. To identify the number of veteran-focused prostate cancer research studies involving primary cell culture that were published over the past 10 years, the following search strategies were used. PubMed: “prostatic neoplasms” [MeSH Terms] AND (“veterans” [Text Word] OR “veterans” [MeSH Terms]) AND (“Primary cell” [Text Word] OR “organoid” [Text Word] OR “patient-derived” [Text Word] OR “biospecimen” [Text Word]) AND (“cell culture” [Text Word] OR “tissue culture” [Text Word]) AND July 1, 2014:June 30, 2024 [Date - Publication]. Embase: “prostate cancer”/mj AND (“veteran” OR “veteran”/exp) AND (“primary cell” OR “organoid” OR “patient-derived” OR “biospecimen”) AND (“cell culture” OR “tissue culture”) AND [July 1, 2024]/sd NOT [July 1, 2024]/sd. Searches were run on July 1, 2024. To identify corresponding studies published in the general population, the same search strategies were utilized except the words veteran and veterans were removed. Search results were imported into Microsoft Excel (RRID: SCR_016137), where conditional formatting was used to identify duplicate results. Two investigators independently retrieved and reviewed the full text of identified studies to determine inclusion. Any disagreements were assessed and resolved through discussion and consensus among the two investigators.

In addition, a review of recently and actively funded research studies within the VA was conducted using publicly available data (https://www.research.va.gov/about/funded_research/). Studies dating back to 2017 were available to review; therefore, all funded studies between 2017 and 2024 were evaluated. After filtering the data to include studies with a prostate cancer focus, a manual review of each study was conducted by two independent investigators. Associated abstracts/grant proposals were manually reviewed for the incorporation of veteran-derived, live primary cells/tissue and subsequently recorded. In similar fashion to the database search, any disagreements were assessed and resolved through discussion and consensus among the two investigators.

### Patients

Veteran interest in donating biospecimens for live cell prostate cancer research was evaluated through analysis of veteran enrollment in an Institutional Review Board–approved blood and tissue donation protocol (2020-0915), which was open at the Madison VA. To be eligible for this study, veterans needed to have a diagnosis of prostate cancer and be receiving care at the Madison VA. Veterans were identified and enrolled in both medical oncology and urology clinics. Written informed consent was obtained from all patients included in the study. Enrolled patients donated up to 50 mL of blood during standard-of-care blood draws. Blood could be collected at multiple time points over patients’ clinical course and was utilized for live cell isolation and culture as well as molecular analysis of circulating genomic and transcriptomic data. Patients undergoing radical prostatectomy were also eligible to donate prostate tissue at the time of surgery for similar analysis.

Data were analyzed for veterans that were approached between May 27, 2021, and August 30, 2024, for enrollment in 2020-0915. Specific endpoints for analysis included the number of veterans who enrolled in the study and the number that declined enrollment over this time period. Demographic information of the enrolled veterans was also assessed. The demographic information included age, self-described race, whether they lived in a rural setting (using rural–urban commuting area codes >1 to define rural per VA policy), whether they received care for a military service-associated condition, and whether they were castrate-resistant. No demographic information was collected on veterans who declined enrollment.

### Analysis of manual and informatics-based screening methods

Two screening approaches were utilized to identify potentially eligible patients for 2020-0915, a manual screening approach and an informatics screening approach. For the manual screening approach, one team member opened patient schedules for all medical oncology providers (i.e., attending physician, nurse practitioner, and oncology fellow) at the Madison VA with clinics the following week. The team member then looked through the charts of all patients on each schedule to identify patients meeting eligibility criteria. Eligible patients were then manually cross-referenced with a list of patients already enrolled in the study or who had refused the study to exclude patients who had already been presented with the study. Each medical oncology provider was then presented with a list of potentially eligible patients in their schedule who were candidates for trial enrollment.

For the informatics approach, a study team member ran a data query in the VA’s Corporate Data Warehouse (CDW) for patients with International Classification of Diseases, Tenth Revision (ICD-10) codes associated with a prostate cancer diagnosis (C61, C79, and Z85.46) who had appointments in medical oncology clinics at the Madison VA for the upcoming week. The patient list generated from the search function was then cross-referenced against a running list of active study participants using logical formulas and conditional formatting in Microsoft Excel to exclude patients who had already been presented with the study. Each medical oncology provider was then presented with a list of potentially eligible patients in their schedule who were candidates for trial enrollment.

Lists of eligible patients were sent to oncology providers in the form of an emailed report that was sent out each week. Each report listed any patients with appointments in the following calendar week that potentially met enrollment criteria. The provider then had the option to present the study to the patient. Whether or not a study was presented to a patient was left purely to the discretion of the provider. If the provider chose to present the study to one of their patients and the patient expressed interest in enrolling, the provider reached out to a clinical research coordinator (CRC) by phone or internal messaging system. The CRC would then come directly to clinic where they would formally present study consent forms to the patient who could then choose to enroll in the study. To compare the time required to screen provider lists and identify eligible patients using the manual and informatics-based approaches, team members recorded the time it took to generate eligible patient lists each week. Data were then analyzed for a 5-week period from May 22, 2022, to June 5, 2022. This 5-week time period was randomly selected for retrospective analysis to remove potential bias and ensure that all necessary clinical information would be available. Recorded data from each approach were then compared.

### Participant surveys

Following at least 30 days of enrollment in 2020-0915, participants were given an optional anonymous survey that inquired about their prior experience in translational research studies, their current experience as an enrolled participant in 2020-0915, and their feelings on the role of translational research in the VA health care system (Supplementary Fig. S1). The seven-question surveys were distributed to participants either in-person during routine follow-up visits or by mail. The survey was administered to veterans enrolled between February 2023 and September 2024. To administer the survey to patients in this study, the 2020-0915 protocol needed to be amended. Therefore, veterans could only receive the survey if they were able to reconsent to the amended protocol. As not all veterans were able to be contacted to reconsent, the number of surveys administered was less than the number of patients enrolled in the study. Participants were instructed that they had the option to complete the survey in order to help our research team better understand their experiences in our studies as well as their perception about biospecimen donation and translational research at the VA. Patients were provided a return envelope with postage to return their surveys by mail. The surveys did not include any patient-identifying information. Survey data were recorded by local research coordinators at the Madison VA and stored on secure VA servers. The surveys were independently developed and did not receive validation for this pilot study.

### Statistical analysis

Descriptive statistical analyses were performed using Prism v10 by GraphPad Software (RRID: SCR_002798). Tables and figures were generated using Microsoft Excel and Prism software.

### Ethics statement

The study was conducted in accordance with the Declaration of Helsinki. Patients were enrolled under Institutional Review Board (IRB)-approved protocols (2020-0915).

## Results

### There is a lack of veteran-focused prostate cancer research that utilizes live primary cells

Live primary cell cultures provide a robust translational platform to advance prostate cancer research for veterans. The initial focus of our study was to evaluate available published literature that (i) focused on prostate cancer in veterans and (ii) included live primary cells as a model system. Our goal was to determine the extent of available publications that leveraged this key resource for prostate cancer research and identify potential areas of need for veteran-derived live cell–based research moving forward. Our search strategy utilized the PubMed and Embase databases, which are comprised of more than 38 million and 45 million citations, respectively. We included all published articles with a central focus on prostate cancer that also included veterans and cell/tissue culture as well as one of the following terms: “primary cell, organoid,” “patient-derived,” or “biospecimen,” anywhere in the text. We limited our search to articles published over the last 10 years to focus our investigation on current prostate cancer studies. Two investigators independently retrieved and reviewed the full text of identified studies to determine inclusion. Disagreements were resolved through discussion and consensus among the two investigators.

**Figure 1.**
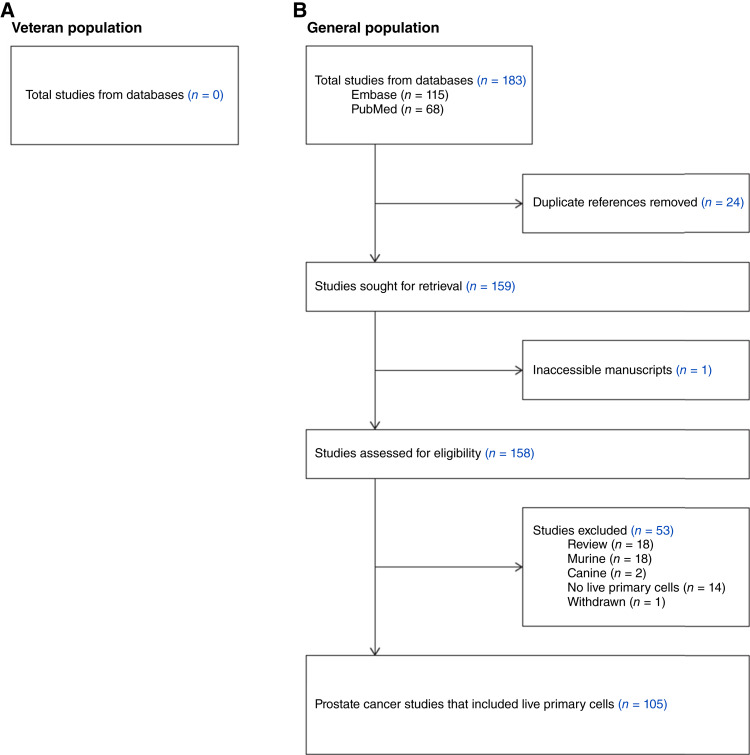
Flow diagram for the literature review. **A,** A total of 0 studies were identified in the last 10 years that incorporated live primary cells in veteran-focused prostate cancer research. **B,** For the general population, search terms identified a total of 183 studies in PubMed and Embase. Of these studies, 78 were excluded for not meeting the inclusion criteria, leaving a total of 105 studies published in the last 10 years that incorporated live primary cells in prostate cancer research.

We anticipated that our search strategy would identify several articles to evaluate. However, we found no published articles in either the PubMed or the Embase databases that met our search terms (Fig. 1A). Although many veterans do receive care outside of the VA, and biospecimen studies that included these veterans would not necessarily have been identified by our research strategy, our findings indicated that there was little to no veteran-focused prostate cancer research publications that included live cell culture of biospecimens published within the prior 10 years. To put these findings in context, we next evaluated the number of articles published in the last 10 years that met all of our search criteria except the requirement that the word veteran(s) be included. This search resulted in a total of 183 articles identified between with two databases (68 in PubMed and 115 in Embase). Following removal of duplicates, we identified 162 unique articles that met our search criteria (Fig. 1B).

Two independent investigators then evaluated each of these articles independently and confirmed that 105 of the published articles (39 abstracts and 66 full manuscripts) were prostate cancer–focused studies that included live cell culture of primary cells. Of these 105 studies, 19 included the use of patient-derived xenografts and 97 included *ex vivo* culture of patient-derived primary cells. This finding demonstrated that although there has not been any published veteran-focused prostate cancer research that included the culture of patient-derived primary cells in the last 10 years, there has been considerable research in the general population that utilized this research strategy and resulted in publication. To ensure that we were not missing any actively funded research that was utilizing live primary cells from veterans that was not yet published, we performed a separate analysis of the VA Office of Research and Development (ORD) website, which provides data on ORD-funded research dating back to 2017. All VA-funded prostate cancer research studies between 2017 and 2024 were manually reviewed for incorporation of live veteran biospecimens. In total, 73 unique studies focused on prostate cancer were identified. Of these 73 studies, only three studies included a description of live primary cell research. Notably, our findings did not provide any information on why there is such a stark contrast in live primary cell prostate cancer research in the general population versus VA patient population. We therefore sought to investigate possible explanations for this discrepancy.

### Veterans are willing to donate biospecimens for primary cell culture research

**Figure 2.**
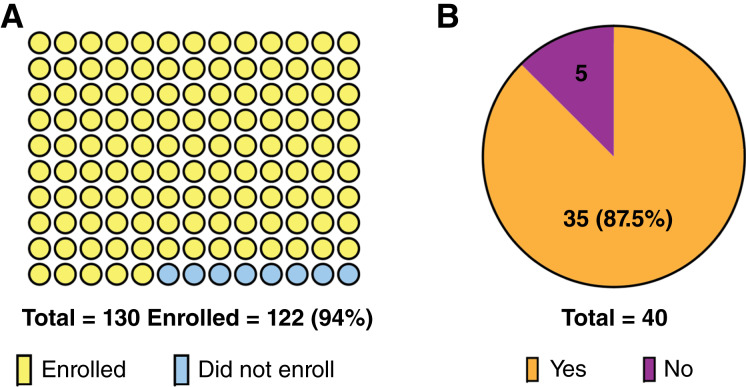
**A,** Bubble chart displaying the enrollment statistics of veterans in our local biospecimen study. Of 130 veterans who presented in the study over the selected time period, a total of 122 veterans enrolled in the study. **B,** Pie chart depicting prior experience in a translational research study in the VA. Nearly 88% of polled veterans who participated in the biospecimen study reported that this was their first time participating in translational research. Only veterans who returned surveys were included in this analysis. In total, 40 veterans completed and mailed back surveys out of the 71 surveys that were distributed.

The lack of published studies utilizing live primary cells could be due to a number of potential factors that could be grouped into two categories: (i) veterans were not interested in donating live biospecimens for translational research studies, or (ii) there were no open studies for veterans to donate their live biospecimens. To investigate veteran interest in donating live biospecimens, we performed an analysis of Veteran enrollment in a translational research study at the Madison VA that included donation of blood and tissue specimens for live cell research. The study involved the collection of blood and tissue samples from veterans receiving care for prostate cancer in the medical oncology and urology clinics at the Madison VA. The primary focus of this study was to isolate live cells from the blood, including circulating tumor cells and immune cell populations, for *ex vivo* culture and modeling of the tumor microenvironment. Between November 19, 2021, and August 8, 2024, 130 veterans with prostate cancer receiving care at the William S. Middleton VA Hospital were identified and offered enrollment in this study. Of those 130 veterans, 122 (94%) consented to participate and enrolled in the study (Fig. 2A).

Based on the high proportion of veterans that enrolled in this study, which only required that they donate biospecimens, we concluded that veterans at our site had a strong interest to donate blood and tissue biospecimens to veteran-focused prostate cancer research. Demographic analysis demonstrated that the demographics of enrolled veterans was overall consistent with the demographics of patients receiving cancer care at the Madison VA (Table 1; Supplementary Table S1).

**Table 1.** Demographics of patients consented to 2020-0915.

Patient demographics	
​	*N* (%)
Age	​
<65 years	9 (7%)
≥65 years	113 (93%)
Race	​
White	104 (85%)
Black	10 (8%)
Declined to answer	4 (3%)
Unknown	2 (2%)
Rural	​
Yes	68 (56%)
No	54 (44%)
Service-connected	​
Yes	78 (64%)
No	44 (36%)
Castrate-resistant	​
Yes	24 (20%)
No	98 (80%)

Most of the veterans were more than the age of 65 years, which would be consistent with the age distribution of prostate cancer. We did not find any clear trend toward increased or decreased participation by race, rurality, or service connection.

### Veterans report satisfaction through participation in a biospecimen protocol

**Figure 3.**
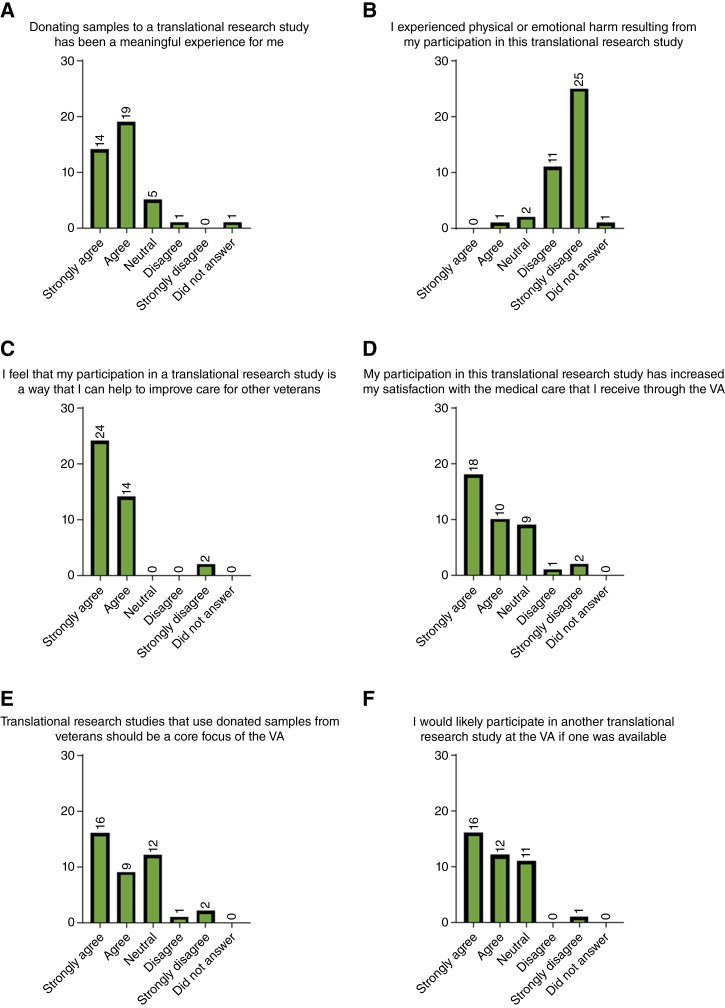
**A–F,** Likert scale questionnaire results among polled veterans.

For the veterans that elected to enroll in our biospecimen collection study, we also wanted to learn more about their prior experiences with translational research as well as their perceptions about translational research following enrollment in our study. We therefore amended our protocol to include a patient survey that was distributed to all patients enrolled in this study. A total of 71 satisfaction surveys were disseminated to patients enrolled in our biospecimen collection study, of which 40 (56%) were completed and utilized for analysis. The majority (*n* = 35, 88%) of respondents noted that this was their first time participating in a translational research study at the VA or any medical institution (Fig. 2B). Following enrollment in our biospecimen donation study, most surveyed patients (*n* = 33, 83%) agreed or strongly agreed that their participation in a translational research study was a meaningful experience (Fig. 3A). Only one participant (2.5%) responded affirmatively (“agree”) that they experienced any physical or emotional harm from their participation in this translational study (Fig. 3B). Importantly, 38 participants (95%) felt that their participation could improve the care of other veterans, and 28 (70%) noted that their enrollment in a translational research study increased personal satisfaction with their medical care at the VA (Fig. 3C and D). In addition, nearly two thirds of respondents (*n* = 25, 63%) felt that translational research studies that use veteran biospecimens should be a core focus of the VA, and 70% (*n* = 28) also indicated that they would participate in a future translational research study at the VA, if it became available (Fig. 3E and F).

### Informatics strategies can reduce time required to screen for eligible patients

As our data demonstrated high enrollment in our biospecimen donation study as well as an overall positive experience in our study, it seemed that veterans (at least at our site) were very willing to participate in live biospecimen donation studies. We therefore investigated whether we could address barriers that hinder the implementation of biospecimen-based research projects from the side of the investigators in order to increase research opportunities for these veterans. One of the major barriers in any study that involves enrollment of human subjects is the process of patient screening (21–23). In order to identify patients to consent for biospecimen donation, investigators and/or study coordinators must manually review large numbers of patient charts to identify patients who meet study enrollment criteria and then compare the list of eligible patients with patients already enrolled. This process is time-consuming, expensive, and highly inefficient (24). Therefore, rather than investing time and resources required for accurate and efficient patient screening, investigators may prefer to utilize more readily available resources, such as cell lines and animal models. To address the logistical challenges of patient screening, we developed an informatics-based screening strategy to identify potentially eligible study participants for trial enrollment at our site. We then tested this strategy using our biospecimen donation study, which was already open at our site.

**Figure 4.**
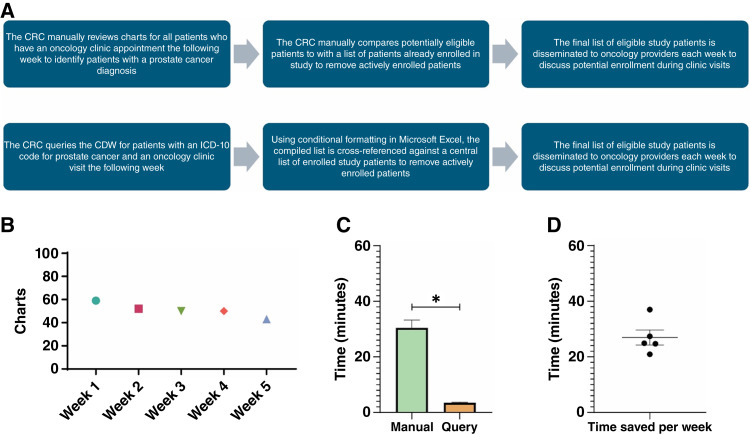
Informatics-based screening strategy. **A,** Manual vs. informatics workflow. **B,** Graph of number of charts per week screened. **C,** Average time per week that it took for coordinator to screen chart manually (manual) compared with coordinator using informatics strategy (query). **D,** Time per week saved by using informatics screening strategy vs. manual strategy. *, *P* < 0.05.

Based on the eligibility criteria for the study, which only required that participants carried a prostate cancer diagnosis and received care at the Madison VA, we queried the VA’s CDW for patients that had upcoming clinic appointments at our site and also had an ICD-10 code associated with a prostate cancer diagnosis (Fig. 4A). The patient list generated from the search function was then cross-referenced against a running list of active study participants to remove patients already enrolled in the study. The finalized list was then disseminated to providers who were able to discuss study enrollment with eligible patients during routine oncology clinic visits and refer patients to appropriate study personnel for enrollment if interested.

To evaluate whether the informatics-based approach saved time compared with a manual chart screening strategy, we collected data on the time it took research personnel to screen provider lists for eligible patients using each approach. We then compared the difference in total time it took to carry out each approach. During the 5-week period of this analysis, a total of 254 patients had attended scheduled oncology clinic appointments at the Madison VA. For the manual screening approach, this resulted in the study team reviewing an average of 50.8 patient charts per week to assess eligibility (Fig. 4B). Over the course of the 5 weeks, the amount of time that it took the team member to perform a weekly chart review ranged from 24 to 41 minutes with an average time of 30.4 minutes per week. Using the informatics approach, it took the team member a range of 2 to 4 minutes per week to identify potentially eligible patients for an average time savings of 27 minutes each week for patient screening during the 5-week assessment period (Fig. 4C and D). Over the course of the 5-week period, this totaled 135 minutes of time saved by using the informatics approach compared with manual chart screening.

We also evaluated the accuracy of the informatics approach to determine how well this screening strategy identified potentially eligible patients. Accuracy was evaluated by comparing the number of patients identified with the informatics approach compared with the manual chart review. This analysis demonstrated that the informatics strategy correctly identified 73 of the 77 (95%) patients with prostate cancer diagnoses scheduled for oncology clinic appointments over the 5-week period. Among the four patients not identified by the search function, two patients had an inappropriate ICD-10 code of D07.5 indicating “carcinoma *in situ* of the prostate”, one patient had an incorrect ICD-10 code denoting essential hypertension (I10), and one patient was missing an ICD-10 code association with their prostate cancer diagnosis. However, even with the few patients that were missed, the informatics screening strategy performed well overall and was therefore implemented for routine patient screening at our site.

## Discussion

It is well established that unique service exposures, such as Agent Orange, are associated with increased prostate cancer incidence, mortality, and adverse outcomes in veterans (3–5). However, the mechanisms by which these specific exposures drive prostate cancer development and whether these exposure-associated cancers should be treated differently than prostate cancers occurring in the general population remains unclear. Primary tissue platforms, such as live cell research using veteran-derived biospecimens, has the potential to shed light on the biology that underlies these veteran cancers to support comprehensive investigation of veteran-focused treatment strategies (18). Unfortunately, our literature review demonstrated that although live primary cells are becoming increasingly utilized for prostate cancer research in the general population, there has been a complete lack of prostate cancer studies that incorporate live cells from veteran donors over the past 10 years. Given the continued and pressing need to improve veteran prostate cancer outcomes, it is critical that efforts are made to incorporate these translationally relevant, live patient-derived models into prostate cancer research to more accurately represent this uniquely high-risk patient population. Furthermore, research that utilizes *ex vivo* primary platforms can help determine whether this approach can lead to meaningful advances in veteran-focused prostate cancer care, which may differ from prostate cancer care in the general population.

We also demonstrated that the donation of biospecimens to support veteran-focused research may be able to provide an important psychologic benefit to veterans. Receiving a diagnosis of cancer can be a highly traumatic experience that can leave patients feeling powerless in their own health outcomes (25). Our survey results in this pilot study suggested that donating live biospecimens to support veteran-directed translational research has the potential to create a meaningful experience for veterans in the context of this challenging disease as well as provide an opportunity for these patients to contribute to the advancement of care for all veterans with prostate cancer. Furthermore, our survey data demonstrated that participation in translational research studies that leverage patient biospecimens could increase veteran satisfaction with the care that they receive from the VA and that veterans feel that this type of research should be a core focus for the VA. Incorporation of more translational research studies that utilize patient-derived biospecimens could therefore be a productive strategy to help veterans deal with the emotional challenges of a cancer diagnosis as well as improve their satisfaction with VA care. However, to fully assess the potential for a psychologic benefit of biospecimen donation, there would need to be a follow-up study utilizing a validate survey that compares the experiences and emotional status of veterans that participate in these types of studies with veterans that do not.

As the data from our study, as well as other studies, have demonstrated that veterans are highly willing to participate in cancer research, we focused on how we could reduce barriers that may prevent investigators from opening trials that rely on veteran-derived live primary cells. We targeted patient screening, which has been noted to be a key barrier to live primary cell research in humans that can take up to several hours and cost more than $300 for a coordinator to screen each patient (21–23). To lessen the burden of patient screening, we implemented an informatics-based screening strategy and demonstrated that this screening approach was generally accurate and decreased the time of weekly patient screening. Similar informatics-based approached have also been leveraged in other contexts and have demonstrated that the utilization widely available, centralized VA resources is a useful strategy for patient screening (26). Our findings in this pilot study support the utility of informatics-based screening as well as provide new contexts in which this screening strategy can optimize patient screening for study enrollment. Although our study did not formally assess whether the time savings associated with this screening strategy contributed to expansion of patient enrollment and study initiation, this was an important first step toward improving efficiency and reducing provider time burden associated with the enrollment process. Our hope is that other investigators will leverage and build upon this screening method to increase the number of live primary cell research studies within the VA.

The expansion of informatics-based patient screening could come through adoption of our screening approach, which has already received funding through the VA ORD to expand the program to the entire Veterans Integrated Service Network (VISN)-12. Alternatively, there are a number of additional informatics programs, resources, and initiatives that can be utilized, including the VA Informatics and Computing Infrastructure, National Artificial Intelligence Institute, and VA Matching Patients to Accelerate Clinical Trials platform (27). Further investigation should also assess management strategies for addressing other existing barriers to enrollment in live primary cell research, including staff/funding shortages, tissue processing, and regulatory burden.

There are multiple potential limitations to our study that should be addressed. Our systematic literature review was limited to published research within the past 10 years to emphasize the current state of research activity regarding veteran-focused primary cell research. This restriction likely led to the exclusion of earlier seminal research studies that utilized donated live cells from veterans (28). These studies were critical to advancing cancer research and provide clear evidence that veterans are willing to contribute to the research field in many ways, including the donation of live primary cells. The findings in our study further support veteran willingness to donate live primary cells as well as the importance of this resource for cancer research.

With regards to the survey assessment of veterans, our analysis relied on responses from veteran participants in a single study at a single institution, which may not be reflective of the veteran population as a whole. To address this limitation, efforts are currently underway to expand our analysis of veteran enrollment in studies utilizing live primary cells using multi-institutional VA networks, including the POPCaP and CRC networks. We are also expanding our informatics-based screening strategy to the entire VISN-12 network through funding from the CRC program. This expansion will include integration of veterans receiving care through community-based practices, which comprise up to 30% of veterans receiving health care in the United States (29). In addition, the survey that was used for this pilot study was independently developed for a specific line of investigation for the researchers. Therefore, this was not a validated instrument, and the conclusions from the surveys should be considered with caution. Efforts are underway to validate this survey prior to utilization in larger future studies.

In our comparison of the manual versus informatics-based screening strategy, only a single research coordinator was utilized for the manual screening. Consequently, this comparison did not factor in the interobserver variability and/or subjective nature of manual screening. Such variables could certainly affect the manual screening data and should be accounted for in future larger studies comparing these two screening approaches. There was also a relative lack of recent demographic data on veteran cancers and related exposures. However, we did not find any data to suggest that the referenced data are no longer accurate.

### Conclusions

There is a notable absence in translational prostate cancer studies that incorporate live cells from veteran donors in recent published literature. Analysis of veteran patients at a single institution demonstrated a high rate of participation in a live primary cell donation study, and enrolled veterans frequently reported positive experiences and increased satisfaction with their care through the VA as a result of their research engagement. Informatics-based screening strategies have the potential to address barriers to initiation and enrollment of veteran patients in translational research studies. Future translational research studies within the VA should therefore consider the utilization of informatics-based strategies to expand research involving live cell biospecimens from veteran donors.

## Supplementary Material

Supplemental Table 1 Supplemental table 1 which displays demographics (race) of patients receiving cancer care at William S Middleton Memorial Veterans Hospital in 2023

Supplemental Figure 1 Supplemental Figure 1 displaying the survey that was distributed to participants in the 2020-0915 study

## Data Availability

Our institutional biospecimen collection protocol does not allow unrestricted public access to veteran data to maintain protection of patient privacy. Therefore, data sharing requests must be submitted to the William S Middleton Memorial Veterans Hospital for review and approval.
