# Supplementary material for: Identifying and Addressing Barriers to Live Primary Prostate Cancer Cell Research in Veterans
Source: Cancer Res Commun. 2026 Jun 29;6(6):1522–30. doi: 10.1158/2767-9764.CRC-25-0776 (PMC13311242; doi:10.1158/2767-9764.CRC-25-0776)
Supplement: Supplemental Table 1 — which displays demographics (race) of patients receiving cancer care at William S Middleton Memorial Veterans Hospital in 2023 [file crc-25-0776_supplemental_table_1_suppst1.docx]

| **Race** | **Percent of total** |
| --- | --- |
| AMERICAN INDIAN OR ALASKAN NATIVE | 0.40% |
| ASIAN | 0.18% |
| BLACK OR AFRICAN AMERICAN | 2.51% |
| DECLINED TO ANSWER | 2.50% |
| MULTIPLE | 0.50% |
| NATIVE HAWAIIAN OR OTHER PACIFIC ISLANDER | 0.23% |
| UNKNOWN | 2.33% |
| WHITE | 91.34% |
| Total | 100.00% |

**Supplemental Table 1. Demographics of patients receiving cancer care at William S Middleton Memorial Veterans Hospital in FY2023**
