# Supplementary figures and images for: Identifying and Addressing Barriers to Live Primary Prostate Cancer Cell Research in Veterans
Source: Cancer Res Commun. 2026 Jun 29;6(6):1522–30. doi: 10.1158/2767-9764.CRC-25-0776 (PMC13311242; doi:10.1158/2767-9764.CRC-25-0776)

**
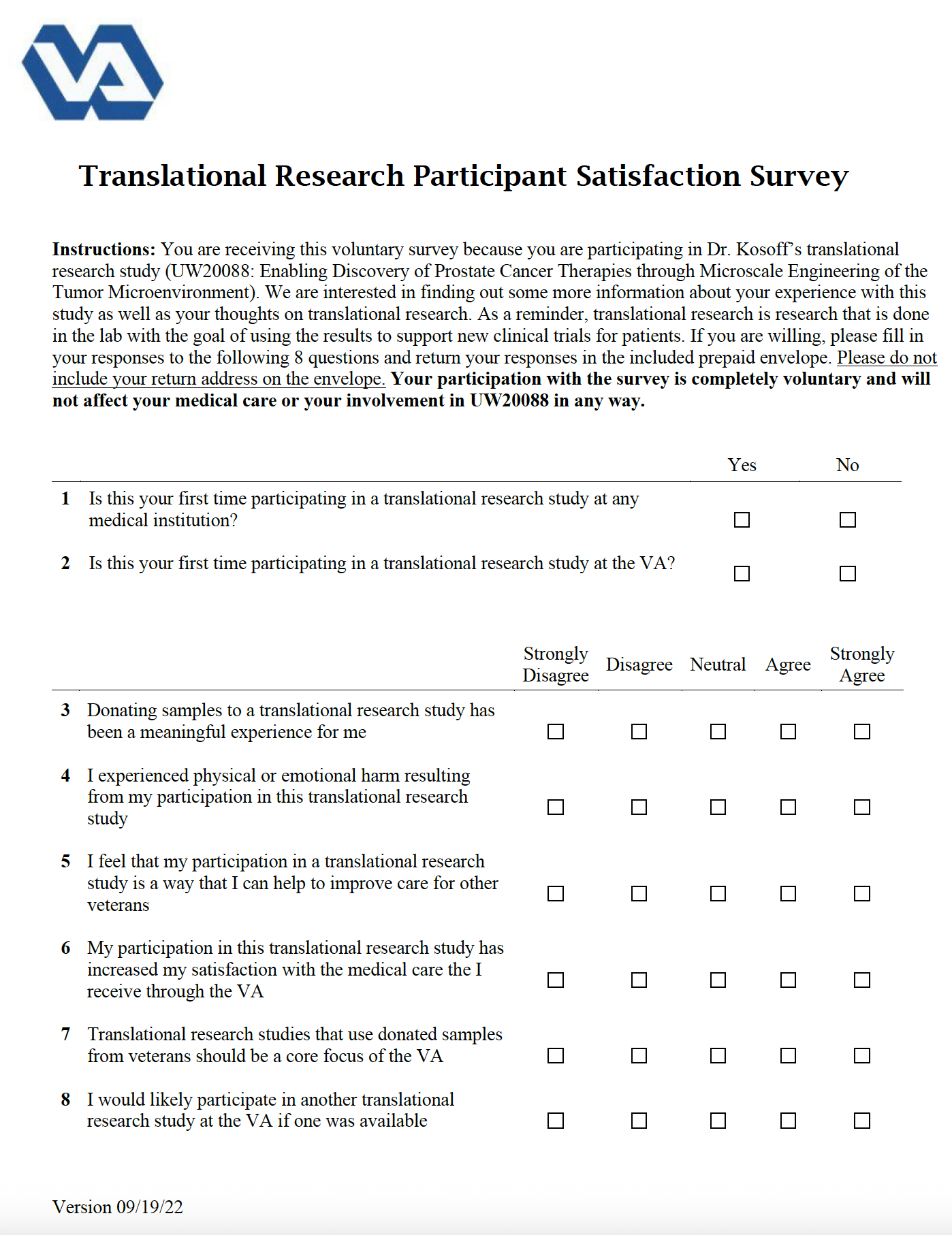
**

**Supplemental Figure 1. Survey distributed to participants of 2020-0915**

Supplement: Supplemental Figure 1 — displaying the survey that was distributed to participants in the 2020-0915 study [file crc-25-0776_supplemental_figure_1_suppsf1.docx]
